# SLAMF receptors negatively regulate B cell receptor signaling in chronic lymphocytic leukemia via recruitment of prohibitin-2

**DOI:** 10.1038/s41375-020-01025-z

**Published:** 2020-08-21

**Authors:** Lisa von Wenserski, Christoph Schultheiß, Sarah Bolz, Simon Schliffke, Donjete Simnica, Edith Willscher, Helwe Gerull, Gerrit Wolters-Eisfeld, Kristoffer Riecken, Boris Fehse, Marcus Altfeld, Peter Nollau, Mascha Binder

**Affiliations:** 1 Department of Internal Medicine IV Oncology/Hematology, Martin-Luther-University Halle-Wittenberg, Halle, Saale Germany; 2 TU Dresden, Biotechnologisches Zentrum, Dresden, Germany; 3 Department of Oncology and Hematology, Bone Marrow Transplantation with Section Pneumology, Hubertus Wald Tumorzentrum-University Cancer Center Hamburg, Hamburg, Germany; 4 Research Institute Children’s Cancer Center and Department of Pediatric Hematology and Oncology, University Medical Center Hamburg-Eppendorf, Hamburg, Germany; 5 Research Department Cell and Gene Therapy, Department of Stem Cell Transplantation, University Medical Center Hamburg-Eppendorf, Hamburg, Germany; 6 Research Department Virus Immunology, Heinrich Pette Institute, Leibniz Institute for Experimental Virology, Hamburg, Germany

**Keywords:** Cancer, Cell signalling

## Abstract

We identified a subset of Chronic Lymphocytic Leukemia (CLL) patients with high Signaling Lymphocytic Activation Molecule Family (SLAMF) receptor-related signaling that showed an indolent clinical course. Since SLAMF receptors play a role in NK cell biology, we reasoned that these receptors may impact NK cell-mediated CLL immunity. Indeed, our experiments showed significantly decreased degranulation capacity of primary NK cells from CLL patients expressing low levels of SLAMF1 and SLAMF7. Since the SLAMF^low^ signature was strongly associated with an unmutated CLL immunoglobulin heavy chain (IGHV) status in large datasets, we investigated the impact of SLAMF1 and SLAMF7 on the B cell receptor (BCR) signaling axis. Overexpression of SLAMF1 or SLAMF7 in IGHV mutated CLL cell models resulted in reduced proliferation and impaired responses to BCR ligation, whereas the knockout of both receptors showed opposing effects and increased sensitivity toward inhibition of components of the BCR pathway. Detailed molecular analyzes showed that SLAMF1 and SLAMF7 receptors mediate their BCR pathway antagonistic effects via recruitment of prohibitin-2 (PHB2) thereby impairing its role in signal transduction downstream the IGHV-mutant IgM-BCR. Together, our data indicate that SLAMF receptors are important modulators of the BCR signaling axis and may improve immune control in CLL by interference with NK cells.

## Introduction

B cell receptor (BCR) signaling plays a critical role in driving proliferation and survival of the malignant clone in chronic lymphocytic leukemia (CLL), supported by the clinical activity of inhibitors targeted toward BCR-associated kinases [1]. Encouraged by the results of clinical trials in relapsed/refractory CLL [2, 3], ibrutinib—an inhibitor of Bruton’s tyrosine kinase (BTK) downstream the BCR—has been recently introduced as front-line treatment of CLL [4–6]. Interestingly, superior activity of BTK inhibition appears to be achievable in CLL with unmutated immunoglobulin heavy chain (IGHV) genes (U-CLL), which is currently deemed to be due to more growth-promoting and less anergic BCR signaling in this subset compared to CLL with mutated IGHV genes (M-CLL) [7, 8]. Yet, it remains essentially unclear what exactly drives this differential sensitivity to BTK inhibition of U- and M-CLL on the molecular level. Also, clinical data on ibrutinib sensitivity of M-CLL suggest that this latter group may be heterogeneous in itself, but no molecular or genetic correlate for this observation has been identified so far [9]. Further insights into the modulation of BCR signaling in U- and M-CLL may therefore elucidate essential pathophysiological clues for more individualized targeting to achieve durable disease control in the majority of patients.

Signaling Lymphocytic Activation Molecule Family (SLAMF) receptors are a group of nine type I transmembrane receptors that are mainly expressed on a variety of immune cells. They are known to be involved in the regulation of NK and T cell responses, mostly by homotypic interactions except for SLAMF2 (CD48) and SLAMF4 that interact with each other [10]. In B cells, this class of receptors has been shown to be expressed in distinct patterns associated with development and activation [11]. A previous study demonstrated that SLAMF1, SLAMF2, and SLAMF7 receptors are rather downregulated on CLL cells as compared to their normal B cell counterpart [12], suggesting that high expression of these molecules may have detrimental (e.g., antiproliferative) effects in the CLL context.

In the work presented here, we provide compelling data that SLAMF1 and SLAMF7 receptors may not only enhance immune control of CLL but also negatively regulate BCR signaling and thereby impact sensitivity towards BTK inhibition in the substantial fraction of patients with SLAMF1 or SLAMF7 expressing M-CLL. This data opens up new perspectives on key pathophysiological mechanisms in this disease that may be exploited for biomarker development to guide treatment choices in CLL.

## Methods

### Patient and sample characteristics

Blood samples of 54 randomly chosen untreated patients with clinical and laboratory features of CLL were collected after informed consent as approved by the ethics commission of the Universities of Freiburg, Hamburg–Eppendorf and Halle (Saale). For the sample size calculation, a time to first treatment (TTFT) difference of 2400 days versus 1600 days in SLAMF1/7 receptor high versus low patients was estimated resulting in a minimum number of 50 patient samples to be included in this analysis to achieve a power of 80%. Cells were purified by Ficoll separation. Age, stage, immunoglobulin mutational status, and cytogenetics (FISH) were recorded (Table 1). In addition, 16 independent CLL samples were freshly used for NK cell experiments.

**Table 1:** Clinical characteristics of the validation cohort.

#	Age	Stage	IgHV	MS	Cytogenetics
141	76	B	IGHV3-48	UM	trisomy 12, del13q
144	61	B	IGHV3-30	UM	del11q23, del13q
147	62	C	IGHV1-69	UM	del11q23, del13q
148	69	B	IGHV1-69	M	del13q
149	57	B	IGHV3-7	M	–
150	67	A	IGHV4-39	M	del13q
154	69	A	IGHV3-72	M	del11q23, del13q
159	80	A	IGHV1-69	M	del13q
160	62	B	IGHV3-30	UM	–
161	67	n.e.	IGHV1-2	UM	del13q, del17p
172	59	A	n.e.	n.e.	del13q
173	54	A	IGHV3-23	M	del13q
176	72	C	IGHV1-69	UM	del11q23, del13q
179	82	A	IGHV3-7	M	–
300	59	A	IGHV3-64	M	del13q, del17p
301	70	C	IGHV3-23	M	trisomy 12
303	75	C	IGHV3-33	UM	–
305	68	A	IGHV4-59	M	del13q
306	65	B	IGHV1-46	UM	del11q23, del13q
307	57	A	IGHV4-34	M	del13q
308	58	A	IGHV1-69	UM	–
322	58	B	IGHV3-11	UM	del13q
338	72	C	n.e.	n.e.	del13q
345	74	A	IGHV2-5	UM	del13q
347	65	A	IGHV7-4	UM	–
348	71	B	IGHV3-9	UM	–
350	76	A	IGHV1-69	UM	del13q, del17p
353	71	A	IGHV3-71	M	del13q
354	78	A	IGHV3-7	M	del13q
355	84	n.e.	IGHV3-30	UM	del13q, del17p
356	68	C	IGHV3-21	UM	del17p
357	54	A	IGHV4-55	M	n.e.
359	43	C	IGHV3-11	UM	del17p
360	66	A	IGHV1-69	M	del13q
362	67	n.e.	IGHV4-61	UM	–
363	75	A	IGHV3-23	M	n.e.
364	68	A	IGHV3-30	UM	n.e.
365	66	A	IGHV3-15	M	n.e.
366	81	n.e.	IGHV3-30	UM	–
368	64	C	IGHV4-55	UM	–
369	84	C	IGHV1-2	UM	–
370	62	A	IGHV3-11	UM	del11q23, del13q
372	78	C	n.e.	n.e.	del13q
373	69	A	IGHV3-30	UM	n.e.
374	63	C	IGHV3-48	M	–
375	77	A?	IGHV2-5	M	del13q
377	72	C	IGHV3-7	UM	–
378	49	C	IGHV1-69	UM	–
379	84	B	IGHV3-53	M	del13q, del17p
380	75	A?	IGHV1-69	M	–
381	49	B	IGHV1-69	UM	–
388	73	n.e.	IGHV4-34	M	n.e.
390	67	C	IGHV1-69	UM	del11q
391	56	A	IGHV4-30	UM	n.e.

*n.e.* not evaluated.

Peripheral mononuclear cells of patients were analyzed by flow cytometry for membrane SLAMF receptor expression with the following antibodies and respective isotype controls: SLAMF1-PE, SLAMF7-AF647, CD5-PC5.5, CD19-PC7, CD45-ECD. Analysis was performed on a Navios Flow Cytometer using the Kaluza software (Beckman Coulter, Brea, California).

### Flow cytometry

All relevant antibodies used in flow cytometry are listed in Supplementary Table S1.

### Generation of genetically engineered CLL sublines

The CLL cell lines MEC-1 and Hg3 were obtained from the German Collection of Microorganisms and Cell Cultures GmbH (DSMZ, Braunschweig, Germany), JVM3 cells were a kind gift of Marco Herling. MEC-1 cells were maintained in IMDM medium, Hg3 and JVM3 in RPMI 1640 medium, both supplemented with 10% fetal bovine serum (Thermo Fisher, Waltham, MA) and 1% Penicillin/Streptomycin (Sigma-Aldrich, St. Louis, MO).

Sequences encoding EAT2, SLAMF1 and SLAMF7 were cloned into Lentiviral Gene Ontology (LeGO) vector LeGO-iC2-Puro+ [13], which was used for CLL cell line transduction. Lentivirally-transduced cells were selected with 1 µg/ml puromycin containing medium. Successful overexpression of SLAMF1 and SLAMF7 was verified by flow cytometry and for EAT2 by western blotting.

gRNAs directed against SLAMF1 (CAGGGAGAGAAACAGCACGA) and SLAMF7 (ATGCAGCACGTACTCCTGGG) were cloned into the lentiCRISPRv2 vector using the BsmBI restriction site as previously described [14]. Non-integrating lentiviruses were produced using the integrase defective packaging plasmid pCMVd8.74-D64.V [15]. After transduction, cells showing a complete knockout were sorted using a FACSAria Illu (BD Biosciences).

IgG switched MEC-1 cells were generated using CRISPR/Cas9 technology as described by Cheong et al. [16].

### Proliferation and cytotoxicity assay of SLAMF receptor overexpressing or knockout CLL cell lines with Ibrutinib

For proliferation and inhibition analyzes, CLL sublines seeded at 0.1 × 10^6^ cells/ml were treated with 1 µM of Ibrutinib (Selleckchem Chemicals, Houston, Texas) or left untreated. After 96 or 120 h, viable cell numbers were measured by trypan blue staining using the Cell Viability Analyzer Vi-Cell XR (Beckman Coulter).

### Ca^2+^ flux measurement

Ca^2+^ flux in transduced cells was measured as described by Schepers et al. [17]. Briefly, cells were loaded with Fluo4-AM (Thermo Fisher) and resuspended in 1 ml PBS containing Ca^2+^. For BCR crosslinking, 5 µg/ml goat-anti-human IgM or IgG Fc antibody (Thermo Fisher, H15000 or H10300) was used, respectively. Fluorescence intensity measurement was performed using a FACSCalibur and the BD CellQuest Pro Software (BD Biosciences).

### RNAseq and pathway analysis

Normalized RNAseq data from 304 CLL patients (EGAS00001000374) was downloaded from the ICGC data portal and analyzed for SLAMF receptor expression levels.

### Biotinylation screen

For the Biotinylation screen, the sequence of a promiscuous Biotin ligase [18] (BioID2) was cloned in frame to the C-terminus of SLAMF1 and SLAMF7 in the respective LeGO-vectors and cells were transduced with the resulting lentiviral particles at low multiplicity of infection. After puromycin selection, biotin was added to the media at a final concentration of 50 µM. After 24 h, cells were harvested, lysed, and subjected to either streptavidin pull down followed by gel electrophoresis and mass spectrometry or immunoblotting of whole cell lysates was performed for visualization of the differential biotinylation patterns.

### Western blot analysis

All relevant primary antibodies used for immunoblotting are listed in Supplementary Table S2, secondary antibodies were: anti-mouse-HRP (HAF007, R&D Systems, Minneapolis, Minnesota) and anti-rabbit HRP (A0545, Sigma Aldrich). Biotinylation was visualized using HRP-Streptavidin (405210, Bio Legend). The signal was red out with the ImageQuant LAS 4000 (GE Healthcare, Chicago, IL).

### Co-immunoprecipitation

Cells lysed in Buffer A (25 mM HEPES-HCl [pH 7.4], 150 mM NaCl, 1 mM EDTA, 10% glycerol, 0.3% SDS) were incubated with anti-PHB2 (12295-1-AP, Proteintech, Rosemont, Illinois), anti-SLAMF1 (MAB1642, R&D Systems) or anti-SLAMF7 (ab237730, Abcam, Cambridge, UK) loaded Protein G dynabeads (Thermo Fisher) o/n at 4 °C on a rotating wheel. Beads were magnetically separated, washed, and boiled in loading buffer followed by gelectrophoretic separation and western blotting using antibodies mentioned in Supplementary Table S2).

### siRNA knock down

For siRNA transfection, we used the Amaxa Nucleofection system (Lonza, Basel, Switzerland). 2 × 10^6^ cells were resuspended in solution V and transfected using program X-01. siRNAs were purchased from Qiagen, AllStars Negative Control siRNA was used as a transfection control and the final concentration was 0.5 µM.

### NK cell assays

NK cell activity was evaluated in co-culture assays with the parental MEC-1 cell line using CD107a as a surrogate marker as described by Alter et al. [19]. NK cells from CLL patients were isolated from peripheral blood via negative selection using RosetteSep Human NK Cell Enrichment Cocktail (Stemcell Technologies, Vancouver, Canada). We assessed the purity of all NK cell fractions used for experiments via FC staining with CD56-PE. The obtained NK cells rested overnight in RPMI + 10% FCS containing 1 ng/ml IL15 (Peprotech, Rocky Hill, New Jersey). On the next day, the cells were mixed with the target cells at a 1:1 ratio in a 96-well plate in the presence of 1.2 µl CD107a-PE/Cy7, IL-15, and 5 µg/ml brefeldin A (Bio Legend, San Diego, California). The ratio was adjusted according to the abundance of residual CLL cells in the preparation. After 5 h_,_ cells were washed, stained with CD3-FITC, and CD56-PE and measured on FACSCalibur using CellQuest software or LSR Fortessa with the FACS Diva Software (all BD Bioscience, Franklin Lakes, New Jersey).

### Statistics

Survival curves were plotted according to the Kaplan–Meier method using PRISM8 (GraphPad, San Diego, CA). Multivariate analyzes were performed using ANOVA with adequate post-hoc tests or Cox regression using R software and the survival package [20].

## Results

### SH2-profiling of CLL samples reveals a distinct SLAMF receptor driven signaling cluster correlating with a favorable clinical course

**Fig. 1:**
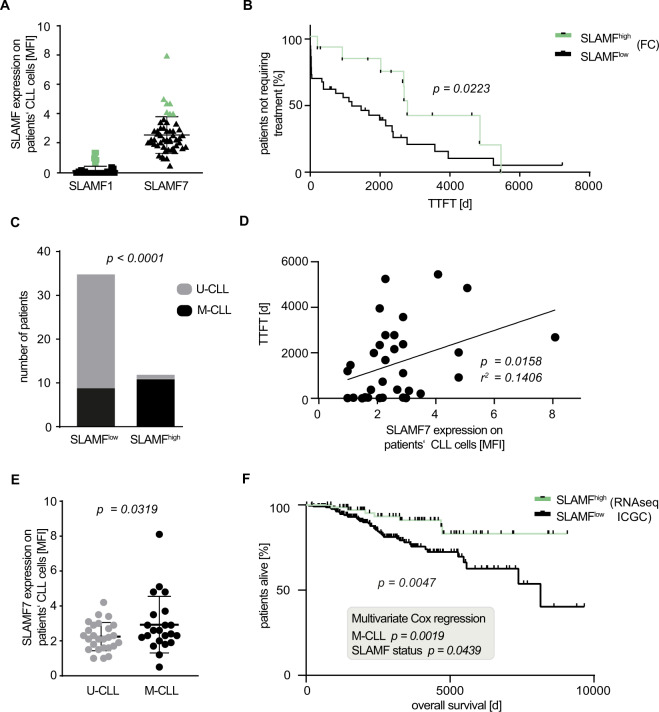
SLAMF1 and SLAMF7 expression in CLL. **a** SLAMF1 and SLAMF7 levels on CLL patients’ CLL cells measured via FC. Values above the upper boundary of SD were considered SLAMF^high^ (green symbols). *N* = 52. **b** TTFT-Kaplan–Meier analysis according to SLAMF status measured in **a**. TTFT-SLAMF^high^ = 2775 days, TTFT-SLAMF^low^ = 1195 days, p = 0.0223 calculated by log-rank test. (SLAMF^high^ = 12; SLAMF^low^ = 39) **c** Correlation between TTFT and SLAMF7 expression on patients’ CLL cells. Pearsons correlation coefficient and statistical significance was calculated, *p* = 0.0158, *r*^2^ = 0.1406. *N* = 33. **d** Association between IGHV mutational status and SLAMF expression. *p* < 0.0001, statistical significance was calculated using Fisher’s exact test. **e** SLAMF7 expression on CLL cells of patients grouped by their IGHV mutational status. *p* = 0.0319, calculated with one-sided unpaired student’s *t* test, error bars represent SD. **f** OS-Kaplan–Meyer analysis of ICGC dataset according to SLAMF1 and 7 expression on RNA level. *p* = 0.0047, calculated by log-rank-test. Multivariate Cox regression was performed to assess independency of variables IGHV and SLAMF status. *N* = 304 (SLAMF^high^ = 75; SLAMF^low^ = 229). FC flow cytometry, SD standard deviation, TTFT time to first treatment, IGHV immunoglobulin heavy chain, OS overall survival.

We previously reported on an unsupervised hierarchical clustering analysis of signaling proteins using Scr-homolgy 2 (SH2) domains as probes [21] in a cohort of 34 patients with CLL. In addition to a signaling cluster essentially driven by the phosphotyrosine Src homology region 2 domain-containing phosphatase-2 (SHP2) [22], we identified a distantly related group of cases displaying high Ewing’s sarcoma-associated transcript (EAT2) -SH2 domain binding. Cases within this latter cluster were characterized by an indolent clinical course with long TTFT as surrogate marker for the aggressiveness of the disease. Yet, the biological significance of the favorable EAT2 SH2 “high” signature remained unclear. EAT2 belongs to the family of SLAM-associated proteins (SAP), which are essential for the signal transduction of upstream SLAMF receptors [23] that modulate innate and adaptive immune responses in various immune cell types [24]. We therefore reasoned that patients in the EAT2 SH2 high cluster may be a subset with high SLAMF receptor levels and SLAMF-related signaling. To confirm our hypothesis, we performed flow cytometry for SLAMF receptors in a second independent CLL validation cohort consisting of 54 patients. These untreated patients encompassed patients with different risk profiles with 42% M- and 58% U-CLL cases (Table 1). From the nine characterized SLAMF receptors, we chose SLAMF1 and SLAMF7 that are expressed in CLL, but on average downregulated in comparison to normal B cells and that carry a cytoplasmic phosphorylation site able to bind to downstream signaling adapters [12]. Indeed, when correlating the expression with clinical data, we found significantly longer TTFT for patients with high expression of one of the SLAMF receptors (defined as less than upper boundary of standard deviation; *p* = 0.0223; Fig. 1a, b, Supplementary Fig. S1). While patients with high SLAMF1 or SLAMF7 receptor levels had a median TTFT of 2775 days (*N* = 12), patients with lower expressions (*N* = 39) had a median TTFT of only 1195 days. Interestingly, in the case of SLAMF7 where we observed a broad spectrum of membrane expression levels, we also found a linear correlation of SLAMF7 membrane positivity with TTFT (Fig. 1c, *p* = 0.0158, *r*^2^ = 0.1406).

A functional link between the BCR and SLAMF receptor expression seemed plausible in that almost all patients from our cohort with high SLAMF receptor expression were M-CLL cases (*p* < 0.0001; Fig. 1d). Also, without using an arbitrary cut-off discriminating between SLAMF high and low CLL cases, we observed quantitative differences in patients with U- and M-CLL with U-CLL cases showing less SLAMF7 density on their CLL cells (Fig. 1e, *p* = 0.0319).

To further validate our findings, we used publicly available RNAseq data of CLL patients provided by the ICGC (EGAS00001000374). We used the same criteria (SLAMF^high^ defined as normalized read counts greater than upper boundary of standard deviation) to divide the 304 patients according to their SLAMF1 or SLAMF7 expression and investigated the subgroups’ survival data. Indeed, the SLAMF^high^ group (*N* = 75) showed significantly longer overall survival than the SLAMF^low^ group (*N* = 229, *p* = 0.0047, Fig. 1f). Interestingly, a very small group of patients with both high SLAMF1 and high SLAMF7 receptor expression (10 of 304 patients) was identified and this small subset showed even superior overall survival (Supplementary Fig. S2A). Moreover, CLL cases with high SLAMF1/7 receptor expression showed a trend towards longer overall survival also within the M-CLL subset (SLAMF^high^ = 36, SLAMF^low^ = 62 patients; Supplementary Fig. S2B). Yet, due to the paucity of IGHV mutational status data in the ICGC dataset, only a Cox regression analysis over the full cohort could clearly show that the SLAMF receptor-related survival difference was not confounded by the mutational status (Fig. 1f). This confirmed that also amongst the M-CLL cases, high SLAMF levels were an independent favorable prognostic marker.

### Effect of overexpression or knockout of SLAMF1 and SLAMF7 on CLL proliferation

We reasoned that SLAMF receptors may directly influence proliferation in CLL, which could explain their prognostic role.

**Fig. 2:**
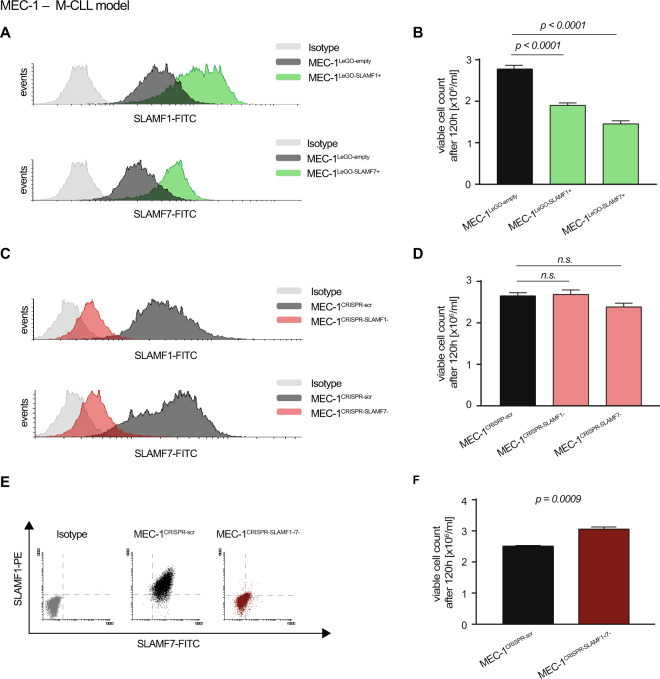
SLAMF1 and SLAMF7 overexpression and knockout in the CLL cell line MEC-1. **a** FC analysis of MEC-1 cells transduced with lentiviral particles coding for SLAMF1 or SLAMF7. **b** Proliferation of SLAMF1 or 7 overexpressing MEC-1 cells after 120 h compared to control cell line transduced with empty vector. *N* = 12. **c** FC analysis of MEC-1 cells after knockout of SLAMF1 or 7 using CRISPR/Cas9 technology. **d** Proliferation of MEC-1cells depleted of SLAMF1 or 7 after 120 h compared to control cell line transduced with a non-targeting (scr) gRNA. *N* = 9. **e** FC analysis of MEC-1 cells after the subsequent knockout of both, SLAMF1 and SLAMF7. **f** Proliferation of MEC-1 cells after knockout of SLAMF1 and SLAMF7 after 120 h compared to control cell line. *N* = 9. Data from independent experiments are shown as mean, error bars represent SEM, statistical significance was calculated by one-way ANOVA and Bonferroni’s post-hoc test. FC flow cytometry, SEM standard error of the mean.

To test this, we used the M-CLL cell line MEC-1 that expresses SLAMF1 and SLAMF7 for overexpression and knockout experiments. In line with the previously established correlation between SLAMF receptor expression on primary CLL cells and an indolent clinical course, we observed lower proliferation rates of the SLAMF receptor overexpressing sublines MEC-1^LeGO-SLAMF1+^ and MEC-1^LeGO-SLAMF7+^ as compared to the control cells MEC-1^LeGO-empty^ (Fig. 2a, b). Since individual knockouts of SLAMF1 or SLAMF7 did not result in significant changes in proliferation (Fig. 2c, d), we hypothesized that these receptors might share redundant functions and one receptor may substitute for the other. We therefore created a double knockout subline (MEC-1^CRISPR-SLAMF1-/7-^) which showed a markedly increased proliferation compared to the control cell line MEC-1^CRISPR-scr^ (Fig. 2e, f).

The proliferative consequences of SLAMF1 and SLAMF7 overexpression could be reproduced in the JVM3 cell line as a different M-CLL model that naturally expresses lower levels of SLAMF1 and SLAMF7 (Supplementary Fig. S3A, B).

Despite the fact that flow cytometry data from our clinical cohort suggested that only a negligible fraction of U-CLL cases shows high expression of SLAMF1 or SLAMF7 receptors (1 of 27 U-CLL cases, as shown in Fig. 1d), we sought to explore SLAMF1 and SLAMF7 overexpression in a U-CLL cellular context (Hg3). Interestingly, Hg3 cells were hard to transduce with SLAMF1/SLAMF7 receptor constructs and sublines resulting from continuous long-term selection pressure showed very low proliferation (Supplementary Fig. S3C, D). This, together with the clinical observation of only few U-CLL cases that highly express SLAMF1/7 receptors suggests that this subset of CLL relies on sufficient downregulation of SLAMF1 and SLAMF7 for survival.

### Modulation of BCR signaling by SLAMF1 and SLAMF7 receptors

**Fig. 3:**
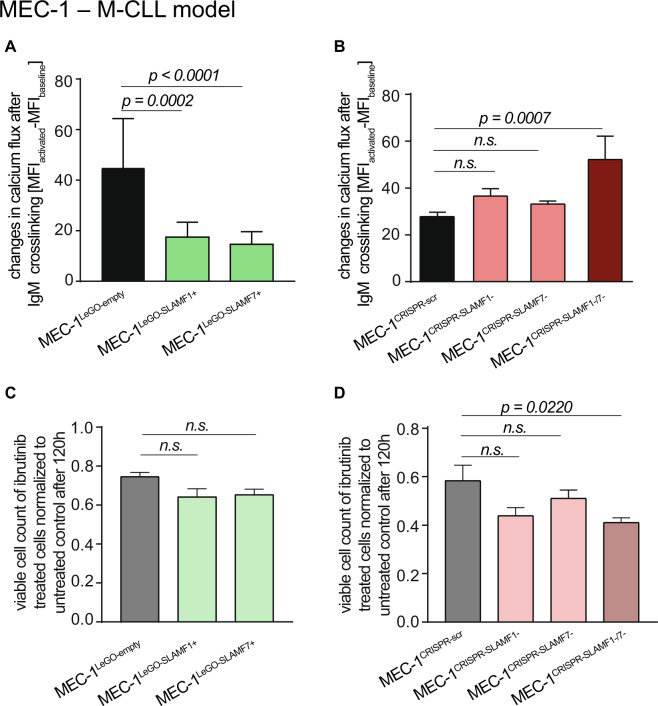
Modulation of BCR signaling by SLAMF1 and SLAMF7 in MEC-1 cells. Cells were stained with FLUO4 and Ca^2+^ flux after stimulation with anti-IgM was assessed via FC in **a** SLAMF1 or 7 overexpressing MEC-1 cells, *N* = 16; **b** in MEC-1 cells after knockout of SLAMF1, SLAMF7 or both. *N* = 8. Cells were treated with 1 µM Ibrutinib and proliferation was measured after 120 h relative to untreated control in **c** MEC-1 cells transduced with empty vector control or overexpressing SLAMF1 or SLAMF7, *N* = 12 or **d** MEC-1 cells after knockout of SLAMF1, SLAMF7 or both, *N* = 9. Data from independent experiments are shown as mean, error bars represent SEM, statistical significance was calculated by one-way ANOVA and Bonferroni’s post-hoc test. FC flow cytometry, SEM standard error of the mean.

Next, we experimentally addressed the question if the antiproliferative effects of SLAMF1 and SLAMF7 receptors may consist in modulating BCR activity since low expression was closely associated with U-CLL. One of the initial steps in B cell activation after BCR engagement is Ca^2+^ flux which subsequently affects numerous cellular functions [25]. Indeed, SLAMF1 or SLAMF7 overexpressing cell lines showed considerably mitigated responses to anti-IgM stimulation (Fig. 3a and Supplementary Fig. S4A, C). Whereas the individual knockouts of SLAMF1 and SLAMF7 receptors did not show any effects on Ca^2+^ mobilization, we observed markedly increased responses to anti-IgM stimulation in MEC-1^CRISPR-SLAMF1-/7-^ (Fig. 3b).

We reasoned that the inhibition of the BCR signaling axis may be less efficient in a cellular background of high SLAMF1 or SLAMF7 receptor expression since BCR signaling appears suppressed by these receptors. To test this, we treated our M-CLL MEC-1 sublines with the BTK inhibitor ibrutinib at half-maximal inhibitory doses. The only cell line showing significantly increased sensitivity toward ibrutinib compared to the respective control cell line was MEC-1^CRISPR-SLAMF1-/7-^ (Fig. 3c, d). These data confirmed the inhibitory effect of SLAMF receptors on the BCR signaling axis. Ibrutinib sensitivity assays performed on our alternative U- and M-CLL models gave very similar results (Supplementary Fig. S4B, D). Of note, we observed not only a high ibrutinib responsiveness in the parental U-CLL Hg3 cell line—well in line with the clinical observation of high ibrutinib sensitivity in U-CLL—but also a markedly decreased sensitivity in the SLAMF1 and SLAMF7 overexpressing Hg3 sublines (Supplementary Fig. S4D).

### Identification of BCR pathway inhibiting mediators downstream of SLAMF1 and SLAMF7 receptors

Based on these findings, we asked how SLAMF receptors modulate BCR signaling in CLL.

**Fig. 4:**
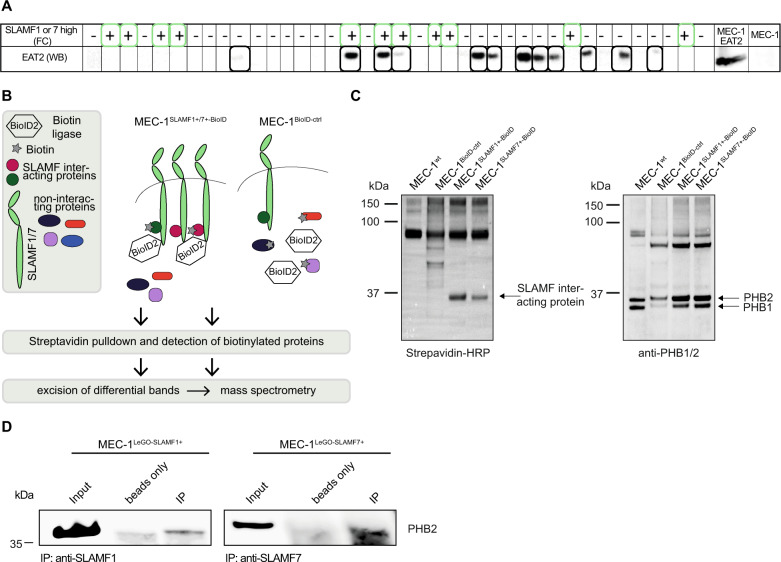
Identification of downstream binding partners of SLAMF receptors in CLL. **a** WB analysis for EAT2 expression in CLL samples from the patient cohort and correlation with SLAMF status. MEC-1 cells overexpressing EAT2 were used as a positive control. **b** Experimental overview of the BioID screen to identify SLAMF1 and SLAMF7 receptor binding partners. **c** WB analysis of whole cell lysates from MEC-1 cells and sublines transduced with BioID2 constructs (either fused to SLAMF1 or 7 or uncoupled as control) incubated with Streptavidin-HRP or PHB1/2 antibodies. **d** Co-immunoprecipitation of SLAMF1 and SLAMF7 in MEC-1 cells. Immunoblotting was done using PHB2 antibody. WB western blot, BioID2 biotin ligase.

Since our screening platform uses SH2 domains provided by signaling molecules or adapters (EAT2, SHP2 etc.) to characterize activated signaling upstream thereof, the expression of the SH2-donating molecule itself in the target tissue is not required—even if the respective SH2 probe shows reactivity. Since SAP family proteins are not uniformly expressed in B cells [26, 27] and our MEC-1 cell line did also not express EAT2 (Fig. 4a), we hypothesized that EAT2 itself may not mediate the SLAMF receptor-related effects in our CLL cohort. To test for EAT2 expression, we randomly selected individual CLL cases with low or high SLAMF1 or SLAMF7 receptor expression levels and subjected these to western blot analysis for EAT2 using MEC-1 cells transduced to express EAT2 as positive control. In line with our assumption, we found no EAT2 expression in the majority of CLL samples (69%) and no correlation with SLAMF receptor status in the few samples positive for EAT2 supporting our hypothesis (Fig. 4a). RNAseq data of MEC-1 cells as well as immunoblotting of CLL samples could also rule out the other SAP family member SH2D1A as the downstream mediator of the SLAMF related effects in CLL as no expression could be detected (data not shown).

It was previously postulated, that SLAMF receptors can signal through inhibitory molecules such as SHP1/2 or SH2 domain containing inositol polyphosphate 5-phosphatase 1/2 (SHIP1/2) in the absence of SAP family proteins [10]. However, our SH2 screens performed using the respective SH2 domains did not show EAT2-like signatures. The only pattern potentially compatible with the EAT2 signature was that of SHP1, but knockdown of this target did not restore proliferation or Ca^2+^ signaling in MEC-1 overexpressing SLAMF receptors (data not shown).

**Fig. 5:**
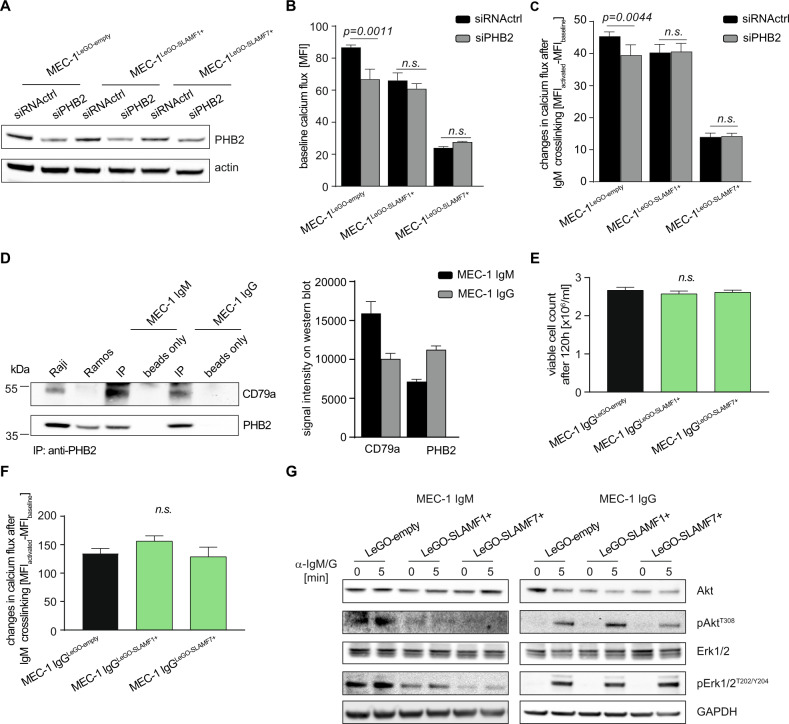
The role of PHB2 in BCR pathway inhibitory effects of SLAMF1 and SLAMF7 receptors in CLL. **a** Representative WB analysis of PHB2 levels 96 h after siRNA transfection as indicated in the MEC-1 sublines. **b** Calcium flux was assessed 96 h after siRNA transfection of MEC-1 sublines baseline and **c** after IgM crosslinking. *N* = 6. **d** Co-immunoprecipitation of PHB2 in IgM and IgG-switched MEC-1 cells. Raji and Ramos cell lysates were used as a positive control for CD79a. For Western Blots, anti-CD79a and -PHB2 antibodies were used. Signal intensities of two independent western blots were quantified using ImageJ. **e** Proliferation was assessed in IgG-switched MEC-1 cells overexpressing SLAMF1 or SLAMF7 compared to empty vector control line after 120 h, *N* = 9. **f** Changes in calcium-flux after IgG crosslinking were assessed in IgG-switched MEC-1 cells overexpressing SLAMF1 or SLAMF7, *N* = 6. **g** Representative Western Blot analysis for Akt and Erk phosphorylation after stimulation with 10 µg/ml anti-IgM/IgG for 5 min in IgM or IgG-swiched MEC-1 cells overexpressing SLAMF1 or SLAMF7 compared to empty vector. Data from independent experiments are shown as mean, error bars represent SEM, statistical significance was calculated by one-way ANOVA and Bonferroni’s post-hoc test. WB Western blot, SEM standard error of the mean, FC flow cytometry.

To molecularly pin down downstream mediators, we finally conducted a biotinylation screen where we coupled a promiscuous biotin ligase (BioID2) at the C-terminus of SLAMF1 and SLAMF7 to selectively biotinylate and identify SLAMF receptor interaction partners (Fig. 4b) [28]. An ~35 kDa biotinylated protein band was visible both in SLAMF1-BioID2 and in SLAMF7-BioID2 overexpressing cells (Fig. 4c). Streptavidin pull down followed by mass spectrometry of excised proteins in the 30–40 kDa range identified prohibitin-2 (PHB2) as a binding partner of SLAMF1 and SLAMF7 receptors in CLL. This interaction was confirmed by co-immunoprecipitation with an antibody directed against SLAMF1 and SLAMF7 receptors (Fig. 4d). PHB2 was initially described as B-Cell Receptor Associated Protein BAP37. The fact that we found a direct interaction between SLAMF receptors and PHB2 strongly suggested to us that this protein was involved in the BCR pathway antagonistic effects produced by SLAMF1 or SLAMF7 overexpression. To address this experimentally, we transfected MEC-1 cells with siRNA specific for PHB2 (siPHB2) to explore the consequences of its knockdown on the SLAMF1/7 receptor induced BCR pathway antagonism. siPHB2 transfection resulted in partial loss of PHB2 expression in all MEC-1 sublines (Fig. 5a). Interestingly, MEC-1^LeGO-empty^ control cells showed lower baseline calcium flux as well as decreased responses to IgM crosslinking after PHB2 knockdown compared to a transfection control (siRNActrl) indicating that PHB2 contributed to intact BCR signaling consistent with prior reports [29, 30]. The BCR antagonistic effects of the PHB2 knockdown were not observed in the SLAMF1/7 receptor overexpressing sublines (Fig. 5b, c). This led us to speculate that in CLL cells SLAMF1 and SLAMF7 receptors recruit PHB2 away from the IgM molecule thereby functionally inducing a PHB2 loss situation leading to impaired BCR signaling and the knockdown of PHB2 could not add up in this scenario. To more specifically study the role of PHB2 in SLAMF receptor-mediated effects on BCR signaling, we performed experiments using an Ig-switch model. Since PHB2 has been shown to associate specifically with the intracellular domain of the IgM-type BCR [31], we created an Ig-switched MEC-1 subline [16] that expresses IgG instead of IgM (Supplementary Fig. S5A). When PHB2 was immunoprecipitated from IgM versus IgG MEC-1 sublines, we found a much lesser amount of CD79a to be co-immunoprecipitated in IgG MEC-1 suggesting that PHB2 only weakly associates with IgG in our model thereby confirming previous work (Fig. 5d). In contrast to the IgM MEC-1 model, SLAMF1 or SLAMF7 overexpression in the IgG subline (successful overexpression shown in Supplementary Fig. S5B) neither impacted cellular proliferation, nor response to IgG ligation in terms of Ca^2+^ mobilization or AKT/ERK phosphorylation (Fig. 5e–g). Together, this suggested that SLAMF1 and SLAMF7 receptors mediate their IgM-BCR antagonism by recruiting PHB2 and thereby disturbing its function in BCR signal transduction.

### SLAMF1 and SLAMF7 status of CLL patients impacts their NK cell degranulation

**Fig. 6:**
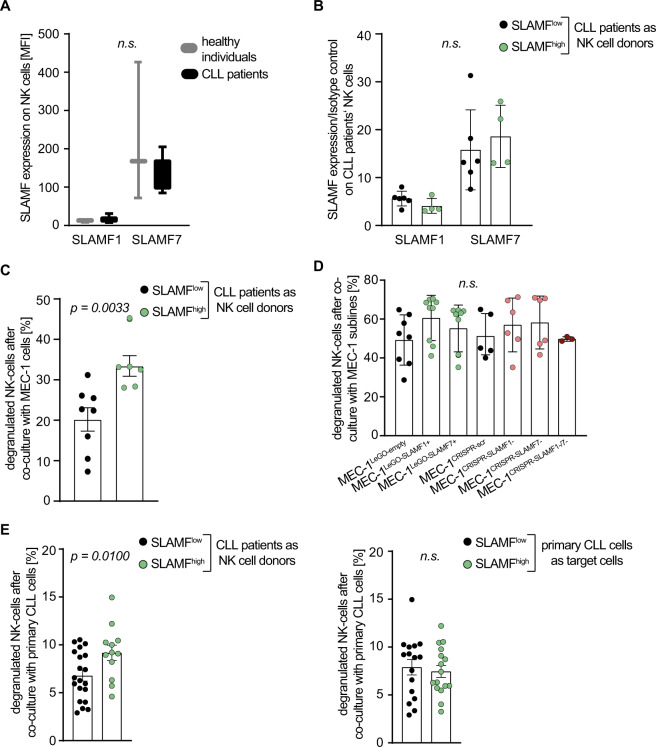
SLAMF receptor expression in NK cell mediated immunity. **a** SLAMF1 and 7 expression on NK cells was measured in CLL patients and healthy individuals via flow cytometry, error bars represent SD. *N* = 3. **b** SLAMF1 and SLAMF7 on NK cells derived from SLAMF^high^ or SLAMF^low^ CLL patients measured by FC. Error bars represent SD, statistical significance was tested with student’s *t* test. *N* = 10 (SLAMF^high^ = 4, SLAMF^low^ = 6). **c** NK cells from CLL patients were co-cultured with MEC-1 CLL cells; *p* = 0.0033. *N* = 14 (SLAMF^high^ = 6; SLAMF^low^ = 8). **d** NK cells from healthy donors were co-cultured with MEC-1 sublines as indicated. In **e** NK cells from CLL patients were co-cultured with primary CLL cells of patients being either categorized as SLAMF^high^ or SLAMF^low^. *N* = 8 (SLAMF^high^ = 3; SLAMF^low^ = 5). The percentage of degranulated NK cells was measured by CD107a expression via FC. Data from independent experiments are shown as mean, error bars represent SEM, statistical significance was tested with one sided, unpaired student’s *t* test or in **d** with one-way ANOVA and Bonferroni’s post-hoc test. SD standard deviation, FC flow cytometry, SEM standard error of the mean.

In addition to the effects of SLAMF receptors on proliferation and BCR signaling via PHB2, we reasoned that SLAMF receptors may promote CLL-directed immune control since these molecules are involved (mostly by homotypic stimulating interactions) in the NK/T cell axis [32]. To this end, we assessed if the levels of SLAMF receptor expression on the CLL patients’ NK cells correlated with the levels on their CLL cells. NK cells derived from patients with CLL showed comparable membrane densities of SLAMF1 and SLAMF7 receptors as compared to healthy individuals and there was no difference in the expression of SLAMF1 and SLAMF7 on the NK cells of CLL patients from the SLAMF^high^ or SLAMF^low^ group. This indicated that SLAMF receptor regulation only occurs in the neoplastic B cell, but not the NK cell compartment in CLL patients (Fig. 6a, b, SLAMF^high^ = 4, SLAMF^low^ = 6 patients). However, when comparing the degranulation capacity of CLL-derived NK cells by measuring CD107a expression we found a significant increase if the donating CLL patient was considered SLAMF^high^ (Fig. 6c, SLAMF^high^ = 6, SLAMF^low^ = 8 patients; *p* = 0.0033). Interestingly, there was no difference in the activity of NK cells from healthy donors when incubated with the genetically engineered MEC-1 sublines showing differential SLAMF receptor levels (Fig. 6d). In line with this, degranulation of CLL-derived NK cells was unaffected by the SLAMF receptor levels of primary CLL cells that were used as target cells (Fig. 6e, SLAMF^high^ NK cell donors = 3; SLAMF^low^ NK cell donors = 5). Together, this indicated differential education of NK cells depending on the SLAMF1/7 receptor status of the respective CLL cells.

## Discussion

Research from the last two decades showing that the BCR is a major driver in CLL has profoundly transformed our therapeutic landscape with the introduction of BCR pathway antagonists in essentially all treatment lines. Yet, we still need to define in which therapeutic sequence and with which combination partners these drugs have to be used in order to achieve optimal clinical results in all patient subsets. These clinical questions require an increased understanding of how CLL cells are driven towards proliferation/survival e.g., by deciphering how BCR signaling—as a key mechanism in malignant CLL cells—is modulated in the different biological subsets of CLL. Also, due to the so far rather disappointing results of common immunotherapy principles in this disease (e.g., checkpoint inhibitors) [33], a novel understanding of CLL-specific immune evasion mechanisms is clearly warranted. These insights will be key for further therapeutic advances in this disease.

In the work reported here, we investigated the biological role of two SLAMF receptors found to have—if highly expressed—a favorable prognostic role in CLL that is independent of other known prognostic markers. We present compelling experimental evidence that high levels of SLAMF1 and SLAMF7 attenuate BCR signaling in the subset of IGHV mutated CLL. According to our data, this “internal” attenuation of BCR signaling may be relevant for ~50% of IGHV mutated cases and our experimental data indicate that it may lead to lesser therapeutic efficacy of the BCR pathway antagonist ibrutinib in this setting. Moreover, our data indicate that CLL cases with a lack of downregulation of SLAMF1 and/or SLAMF7 show more efficient NK cell mediated killing and thereby potentially more CLL immune control.

Previous investigations have already established SLAMF1 as prognostic marker in CLL and mechanistically this has been linked to modulation of autophagy [34–36]. Our data now contribute two independent mechanisms by which these receptors may impact both BCR signaling and NK-mediated CLL cell killing.

The mechanism how SLAMF1 and SLAMF7 attenuate BCR signaling in CLL was not evident for us at first glance. First of all, EAT2—one of the key downstream mediators of SLAMF receptor related effects in NK cells—was mostly found not expressed in CLL [37, 38]. In addition, none of the previously reported SLAMF receptor-interacting signaling molecules (SHP1/2, SHiP1/2) [10] could be confirmed to mediate the BCR pathway antagonistic effects in our CLL models. We therefore chose a biotinylation screen as a biochemical approach to pin down the SLAMF receptor downstream molecules relevant for BCR pathway interference in CLL. This analysis independently identified PHB2 as an interaction partner for both SLAMF1 and SLAMF7. PHB2 has been previously reported to be associated with the IgM BCR [31], but its role in BCR signaling has been largely unexplored to date. Our data now directly links SLAMF1/7 and PHB2 to the IgM-BCR via CD79a as a well-established part of the BCR signaling complex. If these interactions occur via sequential binding or as part of multi-protein complexes remains to be elucidated but co-immunoprecipitation experiments and our biotinylation screen point to a rather close proximity of the involved molecules. Moreover, our PHB2 knockdown experiments suggest that SLAMF1 and SLAMF7 receptors likely recruit PHB2 thereby detaching it from the BCR signaling machinery for which—at least in IGHV-mutant CLL IgM-BCR—this molecule seems to be of high importance. Of note, single versus double knockout and overexpression experiments clearly show that both receptors are able to recruit PHB2 and expression of only one of them is sufficient to induce the observed direct anti-proliferative effects. This aspect should be taken into account when considering diagnostic application. However, we found a very small subcohort (~2–3% of all CLL cases) to be highly positive for both SLAMF1 and SLAMF7. These patients show even better overall survival. In light of the data acquired for this manuscript, we believe that this additional survival benefit is not due to the BCR-related effects of SLAMF receptors reported here. Instead, we hypothesize that the increased overall survival of the double-high expressers could be due to BCR-unrelated effects, e.g., SLAMF1’s role in autophagy [34].

Moreover, since we found the BCR signaling axis to be “internally” attenuated in cell lines with high expression of SLAMF1 or SLAMF7, the observation of relatively low inhibitory effects of BCR pathway antagonists in these lines was not surprising. This finding could also explain the clinical observation that in M-CLL (a subset in which about 50% of cases express high levels of SLAMF1 or SLAMF7), treatment with the BTK inhibitor ibrutinib results in prolonged lymphocytosis and lower tissue cell death rate in comparison to cases of U-CLL while sensitivity to chemotherapy is generally satisfactory [39]. It could imply that M-CLL cases with high SLAMF1 or SLAMF7 expression derive relatively lesser benefit from BTK inhibition as compared to U-CLL (that is predominantly SLAMF1/SLAMF7 low) or the ~50% of M-CLL cases that downregulate SLAMF1/SLAMF7. Future clinical trials should prospectively test this hypothesis since it may help to guide selection of M-CLL patients for upfront chemo(immuno)therapy versus BTK inhibition. Moreover, targeting PHB2 as a combinatorial approach with BTK inhibition may have the potential to deepen responses and should therefore be explored.

The other mechanism by which expression of SLAMF1 or SLAMF7 may impact the favorable outcome of this subset of patients, is their effect on the CLL–NK cell interaction. It is widely accepted that NK cells can recognize and kill CLL cells albeit with decreased efficacy [40–42]. Our own data show that NK cells derived from SLAMF^high^ CLL patients show increased degranulation capacity regardless of the SLAMF receptor expression levels of the target cell they are confronted with. This data suggests some kind of NK cell education rather than a stoichiometric effect of high SLAMF receptor expression on the respective tumor cells in CLL patients with SLAMF^high^ status that leads to more efficient immune control. We recognize that these experiments have been conducted in an artificial co-culture system that lacks many of the immune cell populations present in the CLL lymph node or bone marrow environment. Despite this limitation, we postulate that the SLAMF receptor effect on NK cell killing may contribute to the clinical course of CLL expressing high levels of SLAMF1 or SLAMF7.

Taken together, we show that SLAMF receptors (and downstream PHB2) act as central regulators of BCR signaling and potentially also modulate NK-mediated immune control in CLL. Impact of SLAMF1 and SLAMF7 receptor expression on sensitivity toward BCR pathway inhibitors should trigger evaluation of these receptors as biomarkers of response in future clinical trials.

## Supplementary information

Supplementary tables and figure legends

Supplementary tables 1 and 2

Supplementary Figure 1

Supplementary Figure 2

Supplementary Figure 3

Supplementary Figure 4

Supplementary Figure 5
