# Supplementary material for: SLAMF receptors negatively regulate B cell receptor signaling in chronic lymphocytic leukemia via recruitment of prohibitin-2
Source: Leukemia. 2020 Aug 21;35(4):1073–86. doi: 10.1038/s41375-020-01025-z (PMC8024197; doi:10.1038/s41375-020-01025-z)
Supplement: Supplementary file 1 — Supplementary tables and figure legends [file 41375_2020_1025_MOESM1_ESM.docx]

**Supplementary tables**

**Supplementary table 1:** antibodies used in flow cytometry

**Supplementary table 2:** primary antibodies used in immunoblotting

**Supplementary Figure Legends**

**Supplementary Figure 1: Representative histograms of CLL patient PBMCs stained for SLAMF1 and SLAMF7**

**Supplementary Figure 2: SLAMF1 and SLAMF7 expression and survival in CLL. (A)** OS‑Kaplan-Meier analysis of CLL patients from the ICGC dataset expressing both (SLAMF^double-high^, N=10), only one (SLAMF^high^, N=65) or none (SLAMF^low^, N=229) of the SLAMF1 and SLAMF7 receptors as defined in Figure 1F. p= 0.0126. **(B)** OS-Kaplan-Meier analysis of M-CLL cases in the ICGC dataset according to their SLAMF status. p=0.0722. N=98; M‑CLL‑SLAMF^high^=36, M-CLL‑SLAMF^low^=62.
Statistical significance was calculated by using log-rank test.

OS – overall survival; M-CLL – CLL patients with a mutated immunoglobulin heavy chain gene

**Supplementary Figure 3: SLAMF1 and SLAMF7 overexpression in the CLL cell lines JVM3 and Hg3. (A)** JVM3 cells and **(C)** Hg3 cells were transduced with lentiviral particles encoding SLAMF1 and SLAMF7, overexpression was controlled via FC. Proliferation of SLAMF1 or 7 overexpressing **(B)** JVM3 and **(D)** Hg3 cells cells after 120h compared to control cell line transduced with empty vector. N=9.
Data from independent experiments are shown as mean, error bars represent SEM, statistical significance was calculated using one-way Anova and Bonferroni’s post-hoc tests.

FC – flow cytometry; SEM – standard error of the mean

**Supplementary Figure 4: Modulation of BCR signaling by SLAMF1 and SLAMF7 in JVM3 and Hg3 CLL cells. (A)** JVM3 cells were stained with FLUO4 and Ca^2+^ flux was assessed via FC after stimulation with anti-IgM in SLAMF1 or 7 overexpressing JVM3 cells, N=8. **(B)** Proliferation of JVM3 cells overexpressing SLAMF1 or SLAMF7 treated with 1µM Ibrutinb relative to untreated control after 96h. N=12. **(C)** Hg3 cells were stained with FLUO4 and Ca^2+^ flux was assessed via FC after stimulation with anti‑IgM in Hg3 cells after overexpression of SLAMF1 or SLAMF7. N=12. **(D)** Proliferation of Hg3 cells after overexpression of SLAMF1 or SLAMF7 treated with 1µM Ibrutinib relative to untreated control after 120h. N=12.
Data from independent experiments are shown as mean, error bars represent SEM, statistical significance was calculated by one-way ANOVA and Bonferroni’s post-hoc test.

FC – flow cytometry; SEM – standard error of the mean

**Supplementary Figure 5:MEC-1 Ig switch model (A)** FC analysis of IgG switched MEC-1 cells. **(B**) FC analysis of IgG expressing MEC-1 cells transduced with lentiviral particles encoding for SLAMF1 or SLAMF7.

FC- flow cytometry
