# Supplementary material for: SLAMF receptors negatively regulate B cell receptor signaling in chronic lymphocytic leukemia via recruitment of prohibitin-2
Source: Leukemia. 2020 Aug 21;35(4):1073–86. doi: 10.1038/s41375-020-01025-z (PMC8024197; doi:10.1038/s41375-020-01025-z)
Supplement: Supplementary file 2 — Supplementary tables 1 and 2 [file 41375_2020_1025_MOESM2_ESM.pdf]

Supplementary table 1: Antibodies used in flow cytometry

antigen-fluorochrome	manufacturer	clone	order number
SLAMF1-PE	BD Pharmingen	A12	559592
SLAMF7-AF647	BD Pharmingen	235614	564338
CD5-PC5.5	Beckman Coulter	BL1a	A70203
CD19-PC7	Beckman Coulter	J3-119	IM3628U
CD45-ECD	Beckman Coulter	J33	A07784
CD107a-PE/Cy7	Bio Legend	H4A3	328618
CD3-FITC	Beckman Coulter	UCHT1	IM2181U
CD56-PE	Beckman Coulter	N901	A07788
SLAMF1-FITC	Bio Legend	A-12 7D4	306306
SLAMF7-FITC	Bio Legend	162.1	331818
IgM-FITC	Beckman Coulter	SA-DA4	2040-02
IgG-FITC	Southern Biotech	polyclonal	B30655

Supplementary table 2: Antibodies used in immunoblotting

antigen	manufacturer	clone	order number
EAT2	abcam	polyclonal	ab67417
GAPDH	Santa Cruz	6C5	sc-32233
CD79a	abcam	EP3618	ab79414
PHB2	Santa Cruz	A-2	sc-133094
Akt	Cell Signaling	polyclonal	9272
pAkt (T308)	Cell Signaling	polyclonal	9275
Erk1/2	Cell Signaling	L34F12	4696
pErk1/2 (T202/Y204)	Cell Signaling	polyclonal	9101
