# Supplementary figures and images for: SLAMF receptors negatively regulate B cell receptor signaling in chronic lymphocytic leukemia via recruitment of prohibitin-2
Source: Leukemia. 2020 Aug 21;35(4):1073–86. doi: 10.1038/s41375-020-01025-z (PMC8024197; doi:10.1038/s41375-020-01025-z)

Supplementary Figure 1

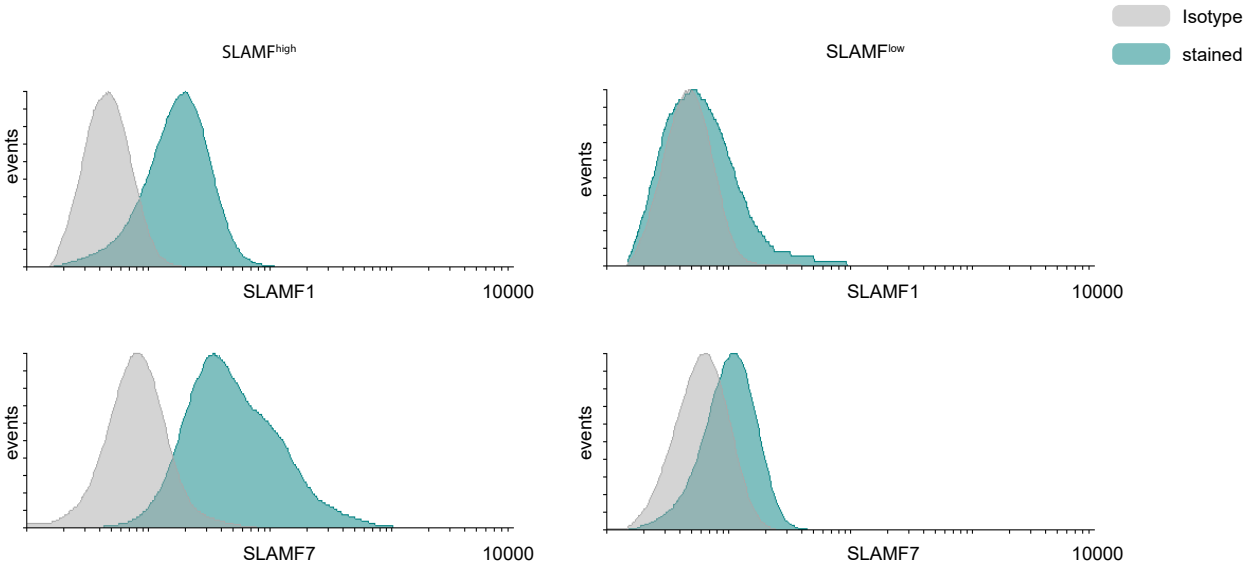

Supplement: Supplementary file 3 — Supplementary Figure 1 [file 41375_2020_1025_MOESM3_ESM.pdf]

Supplementary Figure 2

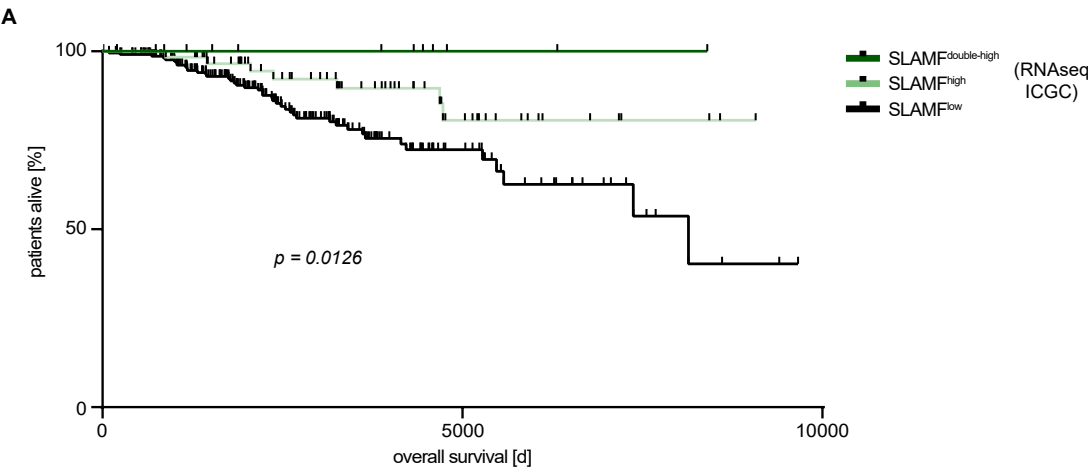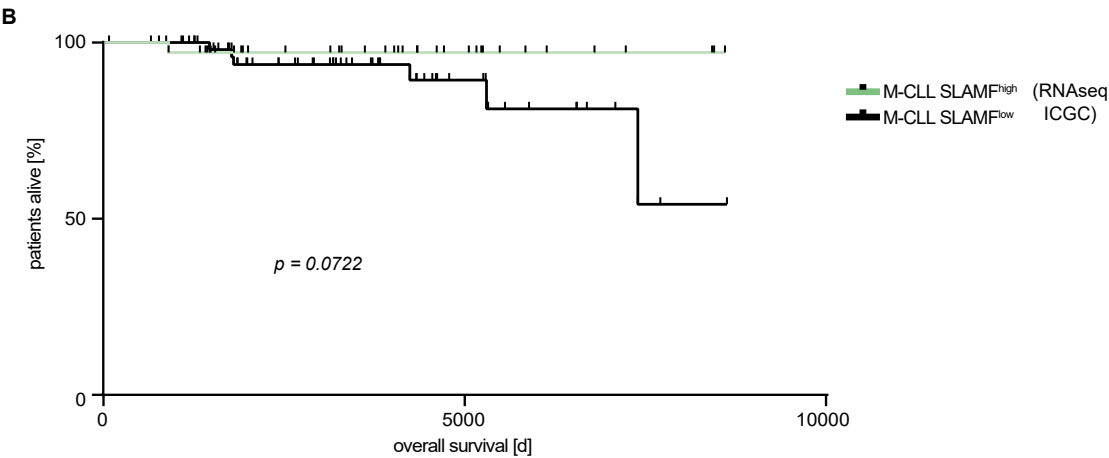

Supplement: Supplementary file 4 — Supplementary Figure 2 [file 41375_2020_1025_MOESM4_ESM.pdf]

Supplementary Figure 3

JVM3 – M-CLL model

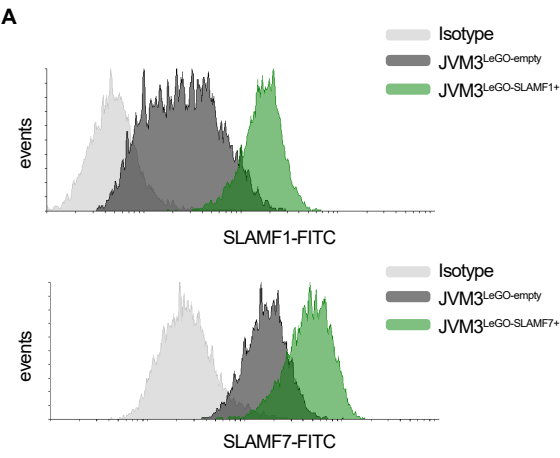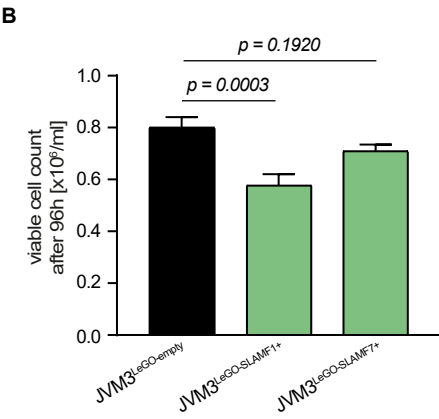

Hg3 – U-CLL model

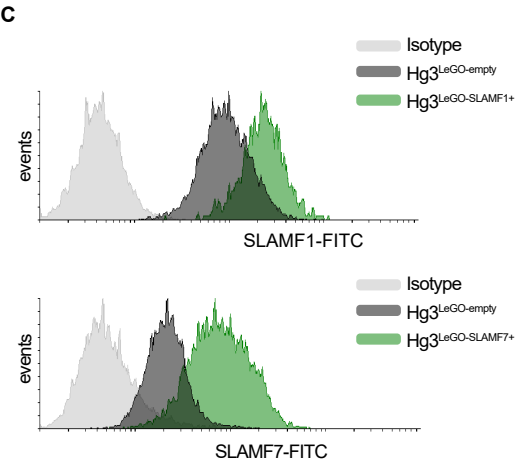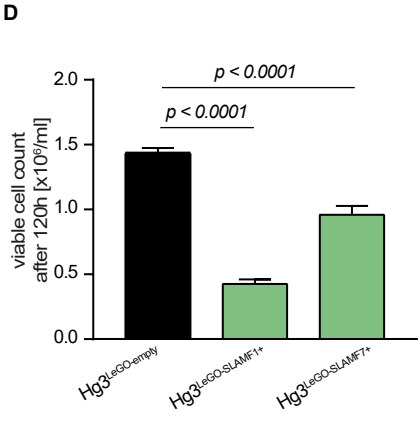

Supplement: Supplementary file 5 — Supplementary Figure 3 [file 41375_2020_1025_MOESM5_ESM.pdf]

Supplementary Figure 4

JVM3 – M-CLL model

A

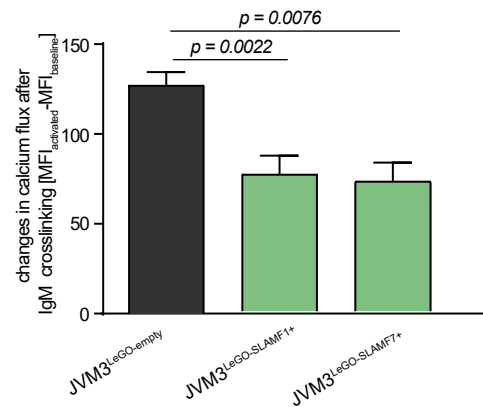

B

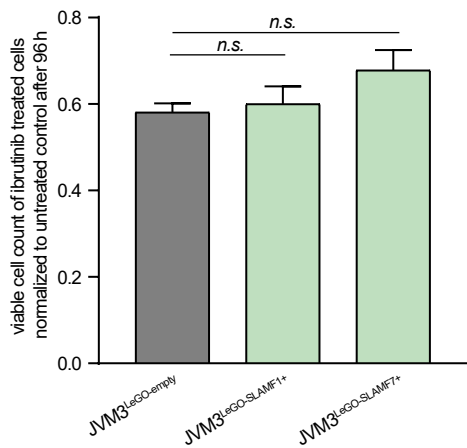

Hg3 – U-CLL model

C

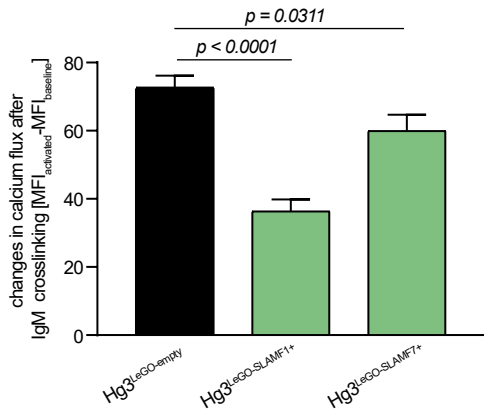

D

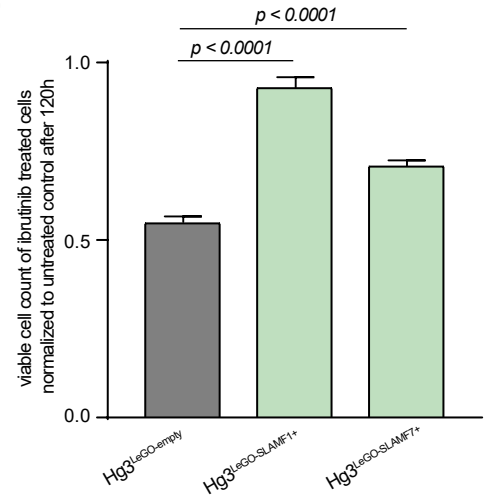

Supplement: Supplementary file 6 — Supplementary Figure 4 [file 41375_2020_1025_MOESM6_ESM.pdf]

## Supplementary Figure 5

MEC-1 – IgG class switch

**A**

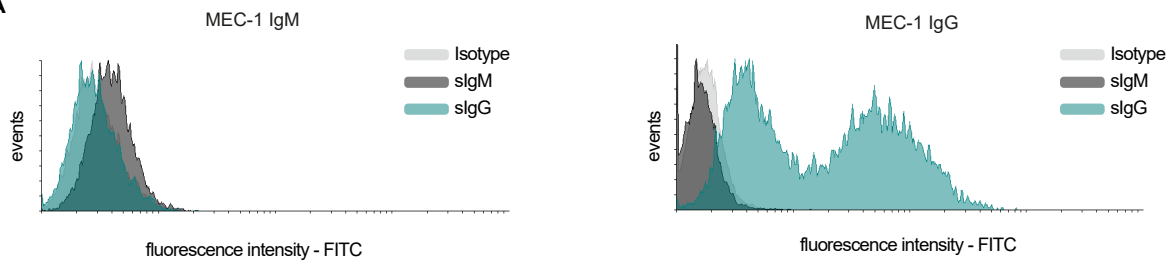

**B**

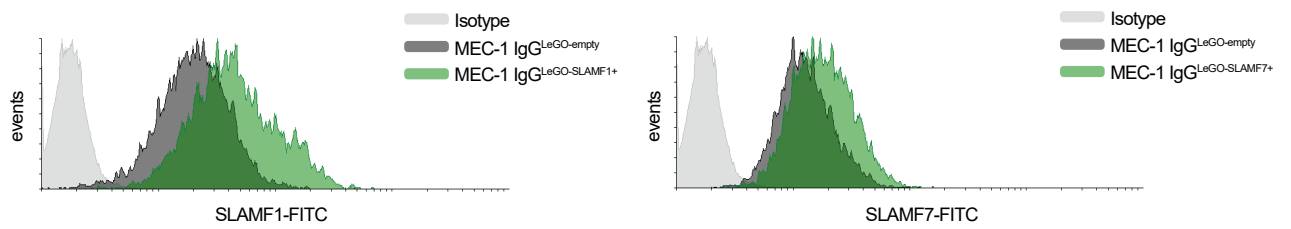

Supplement: Supplementary file 7 — Supplementary Figure 5 [file 41375_2020_1025_MOESM7_ESM.pdf]
